# Risk of Death in Comorbidity Subgroups of Hospitalized COVID-19 Patients Inferred by Routine Laboratory Markers of Systemic Inflammation on Admission: A Retrospective Study

**DOI:** 10.3390/v14061201

**Published:** 2022-05-31

**Authors:** Relu Cocoş, Beatrice Mahler, Adina Turcu-Stiolica, Alexandru Stoichiță, Andreea Ghinet, Elena-Silvia Shelby, Laurențiu Camil Bohîlțea

**Affiliations:** 1 Institute of Pneumophtisiology “Marius Nasta”, 050159 Bucharest, Romania; beatrice.mahler@umfcd.ro (B.M.); alexandru.stoichita@drd.umfcd.ro (A.S.); andreea_bc15@yahoo.com (A.G.); 2 Department of Medical Genetics, University of Medicine and Pharmacy “Carol Davila”, 020032 Bucharest, Romania; gen_og@yahoo.com; 3 Pneumology Department (II), University of Medicine and Pharmacy “Carol Davila”, 020021 Bucharest, Romania; 4 Department of Pharmacoeconomics, University of Medicine and Pharmacy of Craiova, 200349 Craiova, Romania; 5 Department of Cardiology, University of Medicine and Pharmacy “Carol Davila”, 020021 Bucharest, Romania; 6 Scientific Research Nucleus, Dr. Nicolae Robanescu National Clinical Centre for Children’s Neurorecovery, 041408 Bucharest, Romania; silviajdx@yahoo.com

**Keywords:** COVID-19, severity predictors, comorbidity, model, inflammatory markers

## Abstract

Our study objective was to construct models using 20 routine laboratory parameters on admission to predict disease severity and mortality risk in a group of 254 hospitalized COVID-19 patients. Considering the influence of confounding factors in this single-center study, we also retrospectively assessed the correlations between the risk of death and the routine laboratory parameters within individual comorbidity subgroups. In multivariate regression models and by ROC curve analysis, a model of three routine laboratory parameters (AUC 0.85; 95% CI: 0.79–0.91) and a model of six laboratory factors (AUC 0.86; 95% CI: 0.81–0.91) were able to predict severity and mortality of COVID-19, respectively, compared with any other individual parameter. Hierarchical cluster analysis showed that inflammatory laboratory markers grouped together in three distinct clusters including positive correlations: WBC with NEU, NEU with neutrophil-to-lymphocyte ratio (NLR), NEU with systemic immune-inflammation index (SII), NLR with SII and platelet-to-lymphocyte ratio (PLR) with SII. When analyzing the routine laboratory parameters in the subgroups of comorbidities, the risk of death was associated with a common set of laboratory markers of systemic inflammation. Our results have shown that a panel of several routine laboratory parameters recorded on admission could be helpful for early evaluation of the risk of disease severity and mortality in COVID-19 patients. Inflammatory markers for mortality risk were similar in the subgroups of comorbidities, suggesting the limited effect of confounding factors in predicting COVID-19 mortality at admission.

## 1. Introduction

Since the first reported case of coronavirus disease 2019 (COVID-19) in Hubei province, China, in December 2019, this disease, which was initially described as a cluster of pneumonia cases with unknown viral etiology, spread quickly into an ongoing and evolving pandemic that caused millions of infections and deaths worldwide despite the implemented containment measures [1]. Shortly after, the causative agent of the current COVID-19 pandemic, a novel coronavirus named severe acute respiratory syndrome coronavirus 2 (SARS-CoV-2), was identified and sequenced [2].

COVID-19 patients are characterized by heterogeneous symptoms; the most common including fever, cough, fatigue, dyspnea, sputum production, bilateral pulmonary infiltrates, shortness of breath, and headache [3]. Initial symptoms of viral pneumonia can quickly progress in the most critical patients towards acute respiratory distress syndrome (ARDS), sepsis and septic shock, acute cardiac injury, coagulopathy, multiple organ failure, and severe metabolic acidosis [4].

The severity spectrum of COVID-19 ranges from mild or moderate to severe or critical disease leading to death [5]. The clinical classification of patients according to the World Health Organization (WHO) takes into account the correlation between oxygen dependency, disease severity, and mortality [6,7].

The risk of developing a severe outcome or death has been clearly associated with advanced age, male gender, and the presence of multiple chronic medical conditions such as cardiovascular disease, chronic pulmonary disease, cancer, obesity, diabetes, metabolic syndrome, and other factors, including diet and lifestyle [8].

In order to prioritize treatment and consequently prevent disease progression and reduce adverse outcomes by providing early intervention, a fast and accurate prediction method, at admission, of the disease progression towards a critical, severe stage or towards death, is essential.

Many studies have reported early markers of COVID-19 severity that are capable of predicting clinical evolution towards severe complications, such as demographic predictors, laboratory parameters, chest radiographic abnormalities, and other clinical characteristics such as comorbidities or oxygen dependency [9]. Several studies have integrated clinical and paraclinical variables or signs and symptoms, among others, into a clinical prognostic score for the clinical management of COVID-19 patients, to better establish the prognosis of the disease [10,11]. The utility of multivariable machine learning predictive models has also been explored to stratify the patients at admission into predefined groups of disease severity [12,13,14].

Some studies have observed the risk variables without focusing on disease severity, while others have evaluated the distinct risk factors associated with progression to a critical stage [15].

Many studies have found various hematological, biochemical, and inflammatory biomarkers, including white blood cell count (WBC), lymphocyte (LYM) count, platelet (PLT) count, neutrophils (NEU), lactate dehydrogenase (LDH), ferritin, C-reactive protein (CRP), creatine kinase (CK) [16,17,18,19,20], and various relative ratios of different white blood cells, such as the neutrophil/lymphocyte ratio (NLR) and the platelet/lymphocyte ratio (PLR) [21], which can be suggested as predictive markers of COVID-19 disease severity or mortality. Elevated levels of inflammatory markers have been correlated with higher rates of admission to intensive care units (ICUs) and in-hospital mortality [22,23,24]. In addition, dynamic changes in other potential predictors, such as the coagulation function indices including D-dimer, prothrombin time (PT), fibrinogen, and activated thromboplastin time (APTT), have been observed in COVID-19 as signs of intravascular thrombotic complications [25,26].

Although diverse prognostic factors or models for the prediction of unfavorable outcomes in COVID-19 patients have been reported, most of them have required detailed clinical and paraclinical assessments, or have been limited to a narrow analysis, without an absolute extensive assessment of routinely available laboratory tests collected on admission as prediction factors for disease severity or death in subgroups of patients with preexisting comorbidities.

In contrast with these studies, the aim of this research was to assess a set of 20 routine laboratory markers on admission in a group of 254 hospitalized COVID-19 patients in order to identify composite models of predictors capable of accurately stratifying the patients into groups of severity risk defined by the WHO severity score [27].

Furthermore, given the previously reported correlations between different preexisting conditions and COVID-19 severity, and the important association of certain laboratory parameters with a high risk of severe disease, we evaluate whether certain laboratory parameters in our dataset could be associated with the risk of death within each subgroup of comorbidities.

To the best of our knowledge, although different prediction models for COVID-19 severity have been proposed, our study is one of the few advanced analyses to explore whether the performance of compound models exclusively, including routine laboratory variables sampled on admission, is both optimal for classifying the COVID-19 patients according to severity and discriminative for stratifying the patients based on the risk of mortality within the subgroups of patients with comorbidities.

Identifying composite models of paraclinical tests, rather than single markers, available in most laboratories with higher predictability efficiency as early risk factors for unfavorable evolution or death in subgroups of patients with comorbidities is crucial for rapidly optimizing the therapeutic strategies in COVID-19 patients requiring aggressive timely intervention to prevent progression to more serious complications. These models of prediction could be an essential element for the clinical management of COVID-19 disease and the risk reduction of in-hospital mortality.

## 2. Materials and Methods

### 2.1. Study Subjects

This is a retrospective single-center cohort study that included consecutive patients, all diagnosed with COVID-19 and admitted to the Institute of Pneumophtisiology “Marius Nasta”, Bucharest, Romania, between April 2020 and June 2021. The entire study protocol was approved by the Ethics Committee of the Institute of Pneumophtisiology “Marius Nasta”, Bucharest, Romania (No.25657_25658/21.12.2020) in compliance with the Declaration of Helsinki and its amendments. The need for written informed consent was waived because of the retrospective design of the study and the pandemic situation.

Patients enrolled for this study had COVID-19 confirmed on admission by both a positive SARS-CoV-2 real-time reverse transcriptase-polymerase chain reaction (from nasopharyngeal and oropharyngeal swabs) and by computer tomography (CT) scans, independently interpreted by a senior radiologist blinded to the clinical data. Inclusion criteria were adult COVID-19 patients, aged 18 years or more. Exclusion criteria were pregnant women, patients whose clinical data regarding the use of oxygen therapy were missing, patients previously diagnosed with hematological disorders, and patients who, on admission, had severe comorbidities that may have an essential impact on laboratory parameters or patients treated with medication that could alter hematological parameters. No patients were excluded on the basis of sex, ethnicity, or other preexisting conditions.

We classified the clinical severity of our patients at the time of admission and retrospectively according to the WHO classification (WHO/2019-nCoV/clinical/2020.5) by level of disease severity: mild, moderate, severe, and critical [27]. Patients were assigned to clinical groups based on the highest disease severity recorded during hospitalization.

In order to analyze early predictive laboratory variables of severe disease progression, we excluded patients who were hospitalized with severe forms of COVID-19 or who died on the first day of admission. With these criteria, a total of 254 eligible COVID-19 patients were included in the final analysis.

### 2.2. Data Collection

Baseline information, including demographic, clinical, laboratory, and outcome data, was independently extracted from electronic medical records and checked by two trained clinical physicians. Demographic information including age, sex, and smoking status was collected. Clinical symptoms included fever, cough, sputum production, fatigue, dizziness, diarrhea, headache, anorexia, dyspnea, nausea, and shortness of breath. Vital signs included body temperature, heart rate, and oxygen saturation (SpO2). The presence or absence of the following CT findings was included: ground glass opacities (GGO), consolidation, pleural effusion, bronchiectasis, and emphysema. Comorbidities included hypertension, diabetes, chronic obstructive pulmonary disease (COPD), cardiovascular disease, liver disease, renal disease without hemodialysis, obesity, cancer, and cerebrovascular disease.

Additional data, including length of hospitalization, antimicrobial and antiviral treatments, complications, supplemental oxygen (O_2_) by face mask or nasal prongs, noninvasive and invasive mechanical ventilation, as well as hospitalization outcome, was extracted from electronic medical records.

Laboratory parameters recorded at admission were retrieved for each patient, including white blood cell count, lymphocytes, platelet count, hemoglobin (Hb), neutrophils, erythrocyte sedimentation rate (ESR), activated partial thromboplastin time (APTT), alanine aminotransferase (ALT), aspartate aminotransferase (AST), C-reactive protein (CRP), ferritin, D-dimer, total bilirubin, lactate dehydrogenase (LDH), serum creatinine kinase, blood urea nitrogen (BUN), and serum creatinine. Laboratory tests (absolute counts) were used to calculate neutrophil-to-lymphocyte (NLR) and platelet-to-lymphocyte (PLR) ratios, and the systemic immune-inflammation index (SII) using the following equations: NLR = NEU/LYM, PLR = PLT/LYM and SII = PLT × NEU/LYM.

### 2.3. Laboratory Measurements

The hematological measurements at presentation during routine examination and extracted from the electronic medical records were performed using Sysmex XN 1000 (Sysmex, Germany). Hematological quality control materials were analyzed to ensure the quality of data.

The serum biomarkers (ALT, AST, LDH, total bilirubin, serum creatinine kinase, serum creatinine, and BUN), CRP, and ferritin were evaluated using the Beckman Coulter DXC 700 AU (Beckman Coulter, Brea, CA, USA). D-Dimer and activated partial thromboplastin time were measured on ACL TOP 350 platform (Werfen, Bedford, CA, USA).

Additionally, measurement of erythrocyte sedimentation rate (ESR) by the Westergren method was performed for all patients.

### 2.4. Statistical Analysis

Statistical analyses were performed with GraphPad Prism 9.3.0 (GraphPad Software, San Diego, CA, USA) and R (version 4.0.3, GNU General Public License, R Foundation for Statistical Computing, Vienna, Austria).

Data were presented as mean (standard deviation) or median (interquartile range, IQR) values, when reported for continuous variables. Categorical variables were expressed as the number of subjects (n) and percentages (%). Checking for normality was performed using a one-sample Kolmogorov–Smirnov (K-S) test. We used the Mann–Whitney U test (without normal distribution) and paired-samples *t*-test (with normal distribution) to compare among groups. Categorical variables were compared using chi-square test. A two-sided α *p* value of less than 0.05 was considered statistically significant.

Univariate and multivariate analyses were performed to assess the potential risk factors associated with disease severity and mortality in our COVID-19 patients, and the adjusted odds ratio (AOR) was calculated. The variables in the best multivariate model were selected with stepwise selection (Wald). Missing values of laboratory data for univariate and multivariate analyses were replaced via multiple imputation.

The power analysis for our study was performed using G*Power 3.1.9.7 [28] at a 95% confidence level and power factor of 80% for each of the groups. A two-sided *p*-value less than 0.05 was statistically significant. The power test was performed and assuming an alpha level of 0.05, the patients from mild, moderate, severe, and critical groups yielded a power between 70.96% and 99.99% for the different analyses. For example, an a priori assessment to compute the required sample size for WBC (moderate vs. severe, for example), a target of power equal to 80% requires a sample size of 58 moderate patients and 20 severe patients.

To evaluate if the presence of each comorbidity is associated with each laboratory variable, we calculated the *p* values for discharged vs. nonsurviving patients. Additionally, the R ggradar package was used to generate radar plots illustrating the distribution of each laboratory variable in discharged and nonsurviving patients according to baseline comorbidities.

The 95% confidence interval for the odds ratio was calculated for every predictor. The Hosmer–Lemeshow goodness-of-fit test was used to evaluate how well the model fit with data, reflecting the association between predicted and observed risk.

Spearman’s correlation test was carried out to analyze the relationship between laboratory variables, and the R corrplot package [29] was used to plot the correlogram with hierarchical clustering in order to visualize the strength and direction of correlations regarding the laboratory factors influencing the disease severity. The pROC package was further used to plot receiver operating characteristic (ROC) and to calculate the area under the ROC curve (AUC) in order to assess the predictive value of the risk factors on the severity and mortality of COVID-19 patients. We assessed sensitivity, specificity, and AUC (95% CI) for every model.

## 3. Results

A total of 254 eligible patients with COVID-19 who were treated at the Institute of Pneumophtisiology “Marius Nasta”, Bucharest, Romania were included in this retrospective cohort study. In total, there were 141 male (55.5%) and 113 female (44.5%) patients. The median age of our cohort was 52.20 ± 17.13 years. Of the 254 COVID-19 participants, 184 (72.5%) were discharged from hospital and 70 (27.5%) died during hospitalization.

Baseline demographic characteristics, routine laboratory values, clinical symptoms, vital signs, comorbidities, and radiological findings of these patients at admission are summarized and presented in Table 1.

Based on the highest disease severity recorded during hospitalization, the 254 COVID-19 patients were graded according to disease severity into: mild (*n* = 85), moderate (*n* = 98), severe (*n* = 34), and critical (*n* = 37), as depicted in Table 2.

**Figure 1:**
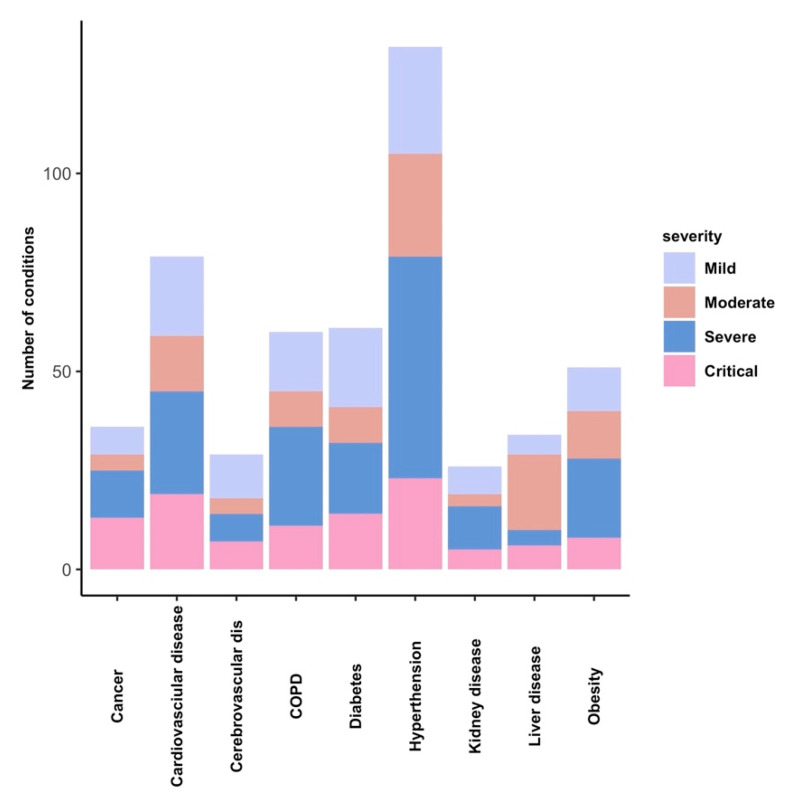
Distribution of preexisting conditions in our group of COVID-19 patients. Number of patients in the disease severity groups illustrated for all subgroups of comorbidities.

Common pre-existing conditions were hypertension (55.1%), cardiovascular disease (28%), diabetes (24.4%), COPD (24%), and obesity (21.3%), as depicted in Figure 1. Of these patients, 28% had no comorbidities, 20.7% had one or two comorbidities, and 33.46% had three or more comorbidities.

**Figure 2:**
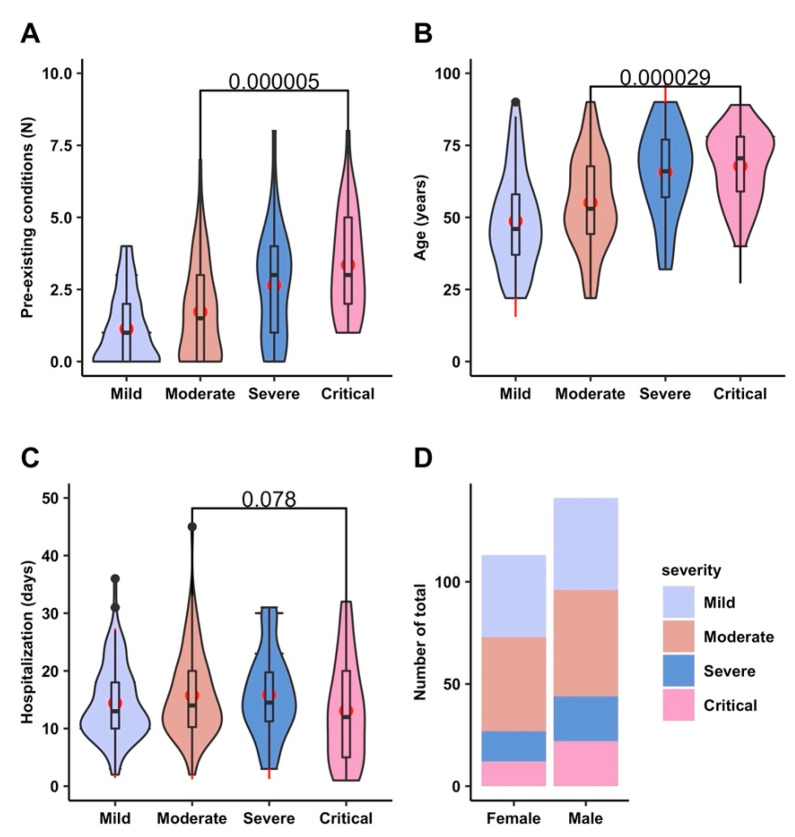
Characteristics of our group of COVID-19 patients. Distribution of preexisting conditions (**A**), age (**B**), and hospitalization days (**C**) in disease severity groups. Percentages of disease severity in female and male patients (**D**). Data are presented as violin plots with medians (**A**–**C**).

On average, nonsevere patients (mild and moderate) had 1.47 preconditions compared to 3 preconditions in severe patients (severe and critical), Figure 2A. Three or more comorbidities were more present in the group of nondischarged patients compared to discharged patients (22.8% vs. 61.43%).

Moreover, severe patients were significantly older than those who were mildly or moderately ill (median age 44.5 and 53, respectively, vs. 70 years, *p* < 0.001), Table 2 and Figure 2B. Males outnumbered females among the COVID-19 patients in our study, but there was no statistically significant difference regarding hospitalization days and sex between the different disease severities (Figure 2C and Figure 2D, respectively).

The baseline laboratory tests on admission of patients with COVID-19 stratified by severity groups (mild, moderate, severe, and critical) are presented in Table 2.

**Figure 3:**
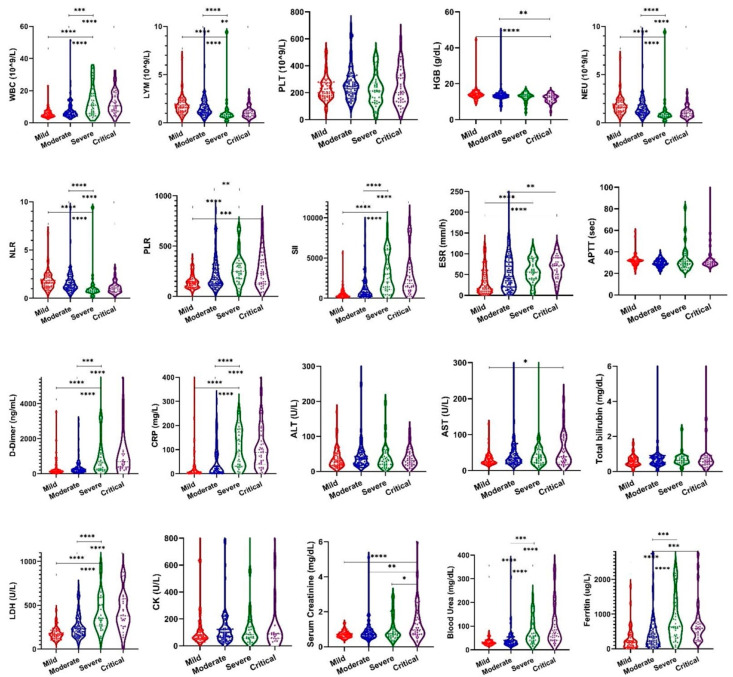
Violin plots showing distribution of laboratory parameter levels on admission. Boxplots indicate median and interquartile range. WHO severity indicates the highest disease severity of the patients during hospitalization. Mild *n* = 85 samples; moderate *n* = 98 samples; severe *n* = 34 samples; critical *n* = 37 samples. The asterisks indicate that the difference between two groups is significant (* *p* < 0.05; ** *p* < 0.01; *** *p* < 0.001; **** *p* < 0.0001).

Patients in severe and critical groups presented significantly elevated values (*p* < 0.0001) of WBC, LYM, PLT, NEU, NLR, PLR, SII, D-dimer, CRP, LDH, BUN, and ferritin as compared to those in mild and moderate severity groups. Specifically, our results have shown that higher values of these laboratory parameters were strongly associated with progression to severe forms, while no significant difference was observed between severe and critical groups, as depicted in the comparative boxplots representing the median laboratory parameters levels at admission stratified according to disease severity groups, Figure 3.

However, at admission, there was no statistically significant difference in sex distribution between these severity groups as compared to age distribution.

In our multivariate analysis, we found three variables independently associated with disease severity, critical or severe, in our groups of COVID-19 patients—age, D-dimer, and LDH—and six variables as mortality risk factors: CRP, ferritin, NEU, NLR, PLR, and SII, Tables S1 and S2 in the Supplementary Material.

The logistic regression analysis showed the best models for combined laboratory predictors for severity and mortality, respectively. Model 3, containing a combination of three routine laboratory predictors and age variables, presented a greater ability to predict disease severity (AUC 0.85; 95% CI: 0.79–0.91) than other models, as illustrated in Table 3. As shown in Table 3, the logistic regression revealed model 7 as the best model for prediction of mortality (AUC 0.86; 95% CI: 0.81–0.91).

**Figure 4:**
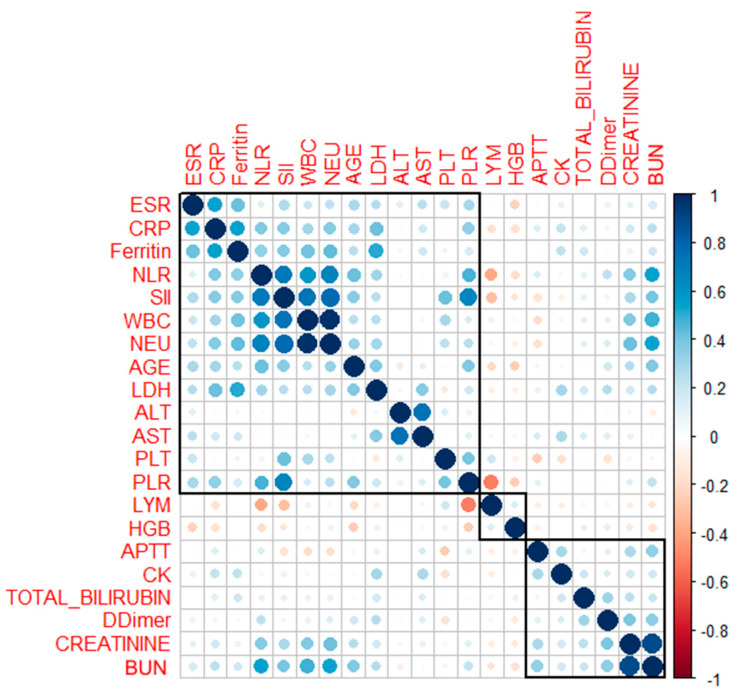
Correlogram with hierarchical clustering of COVID-19 patients. Positive and negative correlations are represented by blue and red dots. The sizes and the shades of the dots reflect the strengths of correlation between pairs of hematological parameters. Colors range from bright blue (strong positive correlation; i.e., r = 1.0) to bright red (strong negative correlation; i.e., r = −1.0). Correlations are ordered by hierarchical clustering with clusters outlined.

Further, we reveal the inter correlations among our laboratory variables at different disease severity levels of COVID-19 by performing Spearman’s correlation analysis. In our group of 254 patients, according to the correlogram of the highest positively correlated parameters (Spearman’s correlation coefficient ≥0.8) were WBC with NEU (ρ = 0.92, *p* < 0.0001), NEU with NLR (ρ = 0.81, *p* < 0.0001), NEU with SII (ρ = 0.86, *p* < 0.0001), NLR with SII (ρ = 0.92, *p* < 0.0001) and PLR with SII (ρ = 0.82, *p* < 0.0001) in the three clusters of correlation, as illustrated in Figure 4.

**Figure 5:**
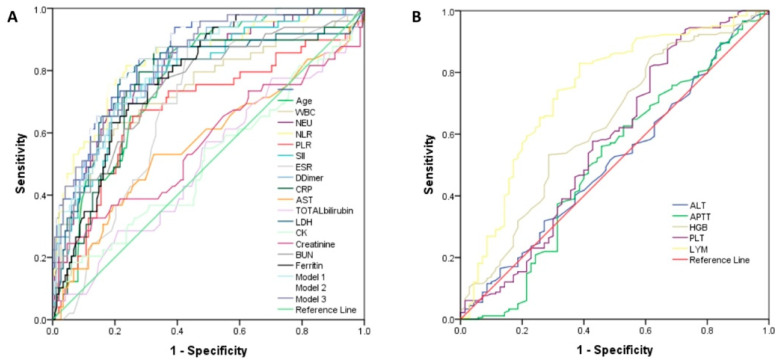
Receiver operating characteristic (ROC) curves for the individual laboratory parameters and models for prediction of disease severity. (**A**) The analysis of AUCs (area under the curve) for age, WBC, NEU, NLR, PLR, SII, ESR, D-dimer, CRP, AST, total bilirubin, LDH, CK, creatinine, BUN, ferritin, and models 1–3; (**B**) ALT, APTT, HGB, and LYM.

**Figure 6:**
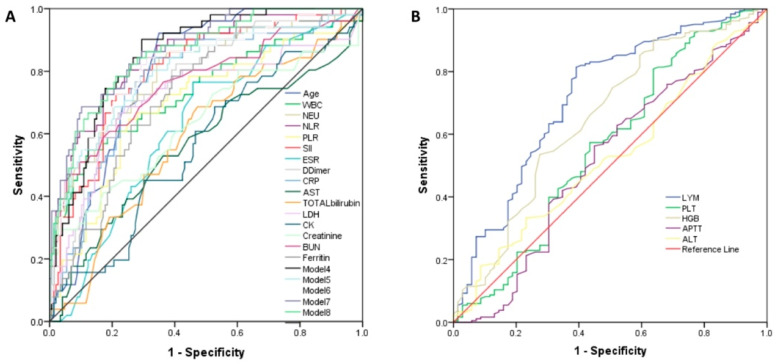
Receiver operating characteristic (ROC) curves for the individual laboratory parameters and models for prediction of mortality in COVID-19 patients. (**A**) The analysis of AUCs (area under the curve) for age, WBC, NEU, NLR, PLR, SII, ESR, D-dimer, CRP, AST, total bilirubin, LDH, CK, creatinine, BUN, ferritin, and models 4–8; (**B**) ALT, APTT, HGB, and LYM.

We next assessed the discriminative power of single laboratory variables in the prediction of disease severity and mortality risk of COVID-19 patients by calculating the area under the receiver operating characteristic (ROC) curves (AUC). A predictor with an AUC between 0.7 and 0.8 was considered fair, while an AUC between 0.8 and 0.9 was considered good. In the analysis of all AUCs among the single laboratory parameters, there were no variables showing a good predictive performance for disease severity and mortality, Figure 5A and Figure 6A (and Tables S3 and S4 in the Supplementary Material).

When examining the use of composite models of laboratory variables, significantly improved AUC values for prediction of disease severity and mortality have been observed. The accuracy of the models, evaluated by AUC as in Figure 5B, suggested model 3 was the best of all proposed ones for prediction of disease severity, while model 7 had the best AUC for prediction of the risk of in-hospital mortality, as illustrated in Figure 6B.

Next, we assessed the routine laboratory parameters collected on admission in each subgroup of comorbidities in relation to COVID-19 death by comparing the discharged patients (*n* = 184) with nonrecovered COVID-19 patients in each of the subgroups of preexisting conditions (see Table S5 in the Supplementary Material). Compared with discharged COVID-19 patients, the levels of WBC, NEU, PLR, SII, D-dimer, CRP, BUN, and ferritin at the time of hospital admission were significantly higher in nonsurviving patients in the subgroup of hypertension (*p* < 0.0001), while the levels of PLR and SII were also significantly higher in nonsurviving patients in the subgroups of obesity and diabetes. When comparing all discharged and deceased patients in the obesity subgroup, our data show that the deceased patients had also significantly higher values of D-dimer and BUN. No significant differences in the other laboratory parameters were observed when comparing all discharged and deceased COVID-19 patients in each of the subgroups of preexisting conditions, excepting WBC in the obesity subgroup, PLR in the cardiovascular subgroup and CRP in the COPD subgroup. COVID-19 patients with any comorbid conditions, considered as a single factor in each group of comorbidities, showed a significant risk of mortality, except for obesity, liver disease, and renal disease. The results in all subgroups of comorbidities are impacted by the effects of other comorbidities considering that three or more comorbidities were more present in the group of nondischarged patients compared to surviving COVID-19 patients, as presented in Figure 2A.

**Figure 7:**
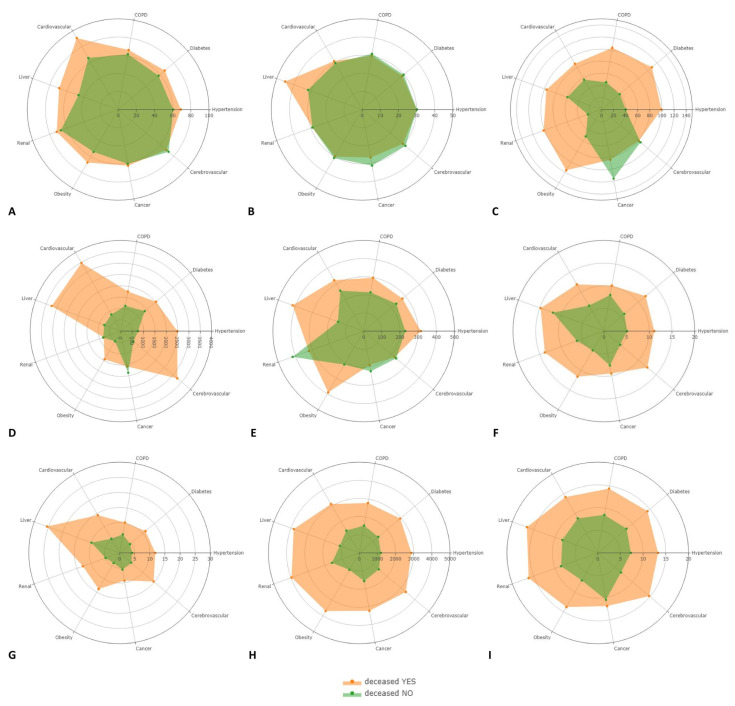
Radar plots illustrating the distribution of scaled values of the selected laboratory parameters in deceased (orange) versus discharged (green) COVID-19 patients comparing each laboratory variable with all comorbidities: Age (**A**), APTT (**B**), CRP (**C**), D-dimer (**D**), LDH (**E**), NEU (**F**), NLR (**G**), SII (**H**), and WBC (**I**).

Further, to observe a potential common pattern of the routine laboratory parameter levels at the time of hospital admission for all subgroups of comorbidity in discharged and nondischarged patients, respectively, we used radar plots to illustrate the distribution of the significant laboratory parameters including age, WBC, NEU, NLR, SII, D-dimer, LDH, CRP, and APTT, as can be seen in Figure 7.

When evaluating the radar plots depicting the laboratory variables specifically associated either with surviving or deceased COVID-19 patients in each comorbidity subgroup, a significantly different distribution profile of the *p* values of CRP, D-dimer, NEU, NLR, SII, and WBC can be observed with each comorbidity. In terms of distribution of age when comparing surviving and deceased patients, the age was similarly represented for each subgroup of comorbidity.

## 4. Discussion

In this single-center retrospective study, we explored the predictive models developed for the severity of disease and mortality using logistic regression, including the common available laboratory parameters for 254 COVID-19 patients at admission. Previous studies have evaluated numerous laboratory biomarkers in predicting the poor prognosis of COVID-19 that can be indicative for inflammatory conditions and signs of organ dysfunction or damage [30,31].

Some patients infected by SARS-CoV-2 can rapidly progress into severe or critical illness characterized by worsening hypoxia, dysfunction of the immune system, tissue injuries leading to systemic inflammatory response syndrome (SIRS), acute respiratory distress syndrome (ARDS), or multiple organ dysfunction (MODS) [32,33]. The common inflammatory markers of the systemic inflammatory response have also been widely documented to be valuable prognostic factors in other pathologies associated with an enhanced inflammatory status [34], such as various types of malignancies, infectious medical conditions, [35,36] and other noninfectious chronic inflammatory diseases [37]. A similar pathophysiology pattern of virus-induced hyperinflammation in COVID-19 has been suggested by several studies for other respiratory viruses such as SARS, MERS (Middle East respiratory syndrome), and influenza A (H1N1) [38], which is reflected by the blood immunological profile of inflammatory markers [39]. Furthermore, preliminary studies have shown that patients with COVID-19 presented a higher value of PLR than influenza A [40,41].

Similar to other existing data, our findings strengthen the correlation between elevated levels of neutrophils, platelet count, D-dimer, CRP, LDH, and ferritin at admission, and unfavorable prognosis in COVID-19 [42].

Significantly higher levels of inflammatory biomarkers in both severe and critical groups indicate that COVID-19 is a potent trigger of inflammatory responses that could be associated with poor clinical outcome [43]. Furthermore, in agreement with this concept, we found increased values of other systemic inflammatory biomarkers such as NLR, PLR, and SII in severe and critical groups compared with those in mild and moderate groups. These relative ratios have been previously shown to independently predictthe progression and prognosis of COVID-19 due to the direct link to changes in lymphocyte count [34,35,36,37,38,39,40,41,42,43,44,45,46]. Our findings are in accordance with large studies that observed that the accentuated depletion of lymphocytes is a marker of disease severity and a characteristic of COVID-19 patients with severe and lethal illness [19,29].

Interestingly, in our cohort of COVID-19 patients, APTT was not closely associated with severe prognosis, although coagulation dysfunction by increased APTT and D-dimer was previously correlated with disease progression towards severe conditions [23,25,47]. Our results are supported by a meta-analysis [24] that has shown normal values of APTT among severe cases. These conflicting results could indicate that the levels of APTT have a direct relationship with the pathophysiology of diseases, later development of coagulation conditions, and heterogeneous behavior of patients regarding the time of presentation at health facilities and clinical severity.

BUN, another biomarker detected as an independent predictor for an unfavorable prognosis in several studies [14,48], was significantly higher at admission in the mild and moderate groups than in the severe and critical groups compared with serum creatinine, another renal marker previously found to be elevated in severe groups [48], which could be explained by the proportions of comorbidities among different studies and clinical severity at admission of COVID-19 patients.

Of note, the impact of COVID-19 on liver enzymes, ALT and AST, was not significantly associated with the severity of the disease, although among our patients increases of AST levels positively correlated with levels of ALT. Regarding severe COVID-19 course, our findings were in line with data from other studies that have shown no significant changes in liver function tests in more severe hospitalized COVID-19 patients [49]. However, a recent systematic review has found a notable role of COVID-19 on liver injury biomarkers suggesting that the prognostic significance of liver function tests for patients with severe conditions could be associated with an elevated host response and aggressive therapy [50]. An explanation for our results is that these potential contributions may specifically depict the biological processes of severe disease that are not clearly reflected by the laboratory parameters recorded at admission for all hospitalized patients.

Models of prediction using laboratory and clinical biomarkers have received considerable attention for early identification of patients at risk of developing severe disease or death from COVID-19 [30,31,51]. In the present study, we considered an approach adjusted by age based on a logistic regression model using a set of twenty routine laboratory parameters. The results of our models showed that signs of disease severity included elevated levels of LDH, D-dimer, creatinine, and BUN, which is consistent with previous studies that analyzed various explanatory models constructed by logistic regression or machine learning [14,52].

LDH, CRP, and D-dimer have been identified by the majority of machine learning models as important risk laboratory parameters linked to COVID-19 disease severity [53,54]. Interestingly, CRP, another nonspecific inflammatory biomarker, was not associated in our logistic regression composite models with severe disease, probably due to the reduced capability of our statistical model to detect complex interactions among attributes. Of note, LDH combined with D-dimer had greater ability to predict disease severity than any laboratory marker alone, with a greater AUC value.

In this study, a composite model identified significant association of death with elevated levels of NEU, NLR, PLR, SII, CRP, and ferritin, providing a significantly larger AUC value than the ones obtained for single laboratory parameters in the prediction of in-hospital mortality. Moreover, the strong positive correlations were observed among the high levels of relative ratios of white blood cell counts, indicating a robust link between the levels of inflammatory markers on admission and poor outcome.

Elevated NLR has been used successfully as an independent prognostic parameter of bacterial infections treatment outcome [34,55], hypertension, heart failure or progression of cancer and response to drug therapy [56]. Endothelial cell death following cellular damage caused by viral infection may be indicated by elevated levels of NLR due to various inflammatory cells, including neutrophils, which in turn leads to multisystemic inflammation by secreting large quantities of cytokines [46,57].

Previous studies reported that NLR, either as an independent predictor or in combination with other clinical parameters [58], helped to predict COVID-19 severity with AUROC values greater than 0.9 [59,60]. Moreover, compared to commonly used inflammatory biomarkers, NLR presented the best predictive value for disease severity across multiple studies among inflammatory markers in COVID-19 patients [61], while others suggested that SII was superior to NLR and PLR in predicting disease severity [62,63]. The potential of PLR to predict the risk of severe disease or ICU admission is controversial [59,64], being optimal only at its peak with only marginal scores in predicting death [45,65].

Our findings suggested for the first time that the combination of these ratios, together with other two inflammatory biomarkers, gave better predictability in the mortality of COVID-19 patients. NLR, PLR, and SII were used as individual parameters to evaluate the inflammatory status and predict outcomes in various conditions associated with systemic inflammation and infection [21]. Increased levels of NLR, PLR, and SII were suggested to be independent risk factors for severity of disease in COVID-19 and considered to be instrumental in the follow-up and diagnosis [44,66]. Likewise, SII was found to have superior predictive ability in comparison to NLR and PLR in COVID-19 diagnosis, while PLR, due to its capability to reflect the degree of cytokine release, was proposed as a predictor for severe COVID-19 [67].

To substantiate a potential casual association between the alterations of laboratory biomarkers at admission and the confounding effects of comorbidities exacerbated by infection with SARS-CoV-2, we further explored the correlation of each laboratory parameter within different comorbidity groups with the risk of mortality in order to identify patterns of variation for each laboratory parameter specifically attributed to two or more comorbidities that are superimposed on the response to COVID-19 infection.

Multiple studies have documented the association between preexisting comorbidities and the risk of mortality [68,69]. Hypertension, cardiovascular disease, diabetes, and obesity are the dominant risk factors for mortality in COVID-19 patients [70,71]. In addition to these, chronic obstructive pulmonary disease (COPD), liver disease, chronic kidney disease, and cancer were observed as preexisting conditions among fatal cases of COVID-19 patients [72]. In line with these meta-analyses, our study identified that hypertension, cardiovascular disease, diabetes, and obesity were the most prevalent among COVID-19 patients. Moreover, compared with the discharged COVID-19 patients, nondischarged patients were more likely to have three or more comorbidities, which is consistent with other previous results showing that patients with multiple morbidities are at the highest risk of developing complications leading to death [73,74].

The precise influence of each of these confounding factors on laboratory parameters at admission, complicated by the systemic inflammation induction effect of COVID-19 is difficult to discriminate in subgroups of comorbidities. Nonetheless, we observed that elevated levels of PLR and SII are significantly associated with mortality in the hypertension, cardiovascular disease, diabetes, and obesity comorbidity subgroups, while higher values of D-dimer and WBC were associated with death in the hypertension and diabetes subgroups. Furthermore, CRP presented significantly elevated values in the hypertension and COPD subgroups. Interestingly, LDH was shown not to be associated with higher risk of mortality in any of the comorbidity groups, which agreed with a previous report that showed no confounding effect of the comorbidities on the association between LDH levels and mortality in patients with COVID-19 [75]. In concordance with these observations, higher levels of various laboratory parameters have been independently related to specific comorbidities in COVID-19 patients in previous reports [72,76]. Thus, for example, although a higher level of D-dimer has been observed in diabetic patients [77], this could also be a consequence of the higher prevalence of kidney and heart comorbidities in diabetic patients.

Our data highlight that some levels of laboratory parameters could be better associated with the risk of mortality for specific subgroups of comorbidities, although a clear distinctive effect of each comorbidity is hard to predict considering the coexistence of multiple comorbidities.

The amplitude of a common inflammatory response to SARS-CoV-2 infection is probably linked to the mortality risk in our subgroups of comorbidities.

Moreover, it is worth noting that, when using a radar plot to illustrate the separate laboratory parameters in patients in the surviving group versus deceased patients in each subgroup of comorbidity, we observed a set of inflammatory markers that had an identical pattern of elevated levels in all comorbidity subgroups, including CRP, D-dimer, NEU, NLR, SII, and WBC. Taken together with previously published results, our findings indicate that it is hard to optimize a mortality risk stratification for each comorbidity subgroup based on laboratory parameters due to the potential multifactorial contributions, although some laboratory parameters are organ-specific and better predict complications and tissue/organ injuries. Nonetheless, our data suggest an essential inflammatory response superimposed on the effect of preexisting conditions crucial for the evaluation of death rather than a specific confounding effect of comorbidities reflected by laboratory parameters at admission, which could be reflected by complications in later stages of COVID-19 disease.

Several approaches have been applied to predict the poor prognosis of COVID-19 patients, including the most commonly used scores for assessing multi-organ dysfunction, sepsis and septic shock such as Sequential Organ Failure Assessment (SOFA), quick SOFA (qSOFA), or Acute Physiology and Chronic Health Evaluation (APACHE II) [78,79,80] in patients during their stay in the intensive care unit (ICU). These score systems are based on a fixed set of physiological factors for evaluating the major organ functions not specifically designed for COVID-19. The evaluation of the SOFA score in severity prediction for in-hospital mortality has produced conflicting results showing either significantly higher scores in the group of patients with severe COVID-19 [79] or no relevant changes; this may be explained by the design of the score, which does not include specific factors associated to mortality in patients with COVID-19 pneumonia requiring mechanical ventilation [78]. In line with these results, other scores such as the 4C mortality score, comprising supplementary variables for computation, have shown a better prediction of poor prognosis as compared to SOFA [81]. In our opinion, compared to the prognosis of COVID-19 severity with scoring systems, the prediction utility of the published models of prognosis based strictly on laboratory parameters is not limited by the clinical data which might be unavailable for nonsevere patients at admission [82]. Moreover, such models could be more appropriate for prediction of all clinical outcomes considering that multiple-organ damage is more pronounced in nonsurvivors and the individual relevance of the components of risk scores in the prediction of mortality, such as the levels of SaO2 at admission [57,82].

There are some potential limitations of our study. First, this is a single-center retrospective study that suffers from the usual limitations such as bias in selection and increased impact of the generalizability of data. Second, the sample size is relatively small, which might affect the reproducibility of our data. A multicenter, prospective study is needed to further understand the prognostic utility of the laboratory markers in our model. Third, we did not test other routine biomarkers due to data unavailability for the majority of our patients, but the goal of the present study was to include those biomarkers that are easily and commonly available. Despite these limitations, this is a strong study, recording accurate and detailed data on comorbidities, allowing us to evaluate the risk of mortality within individual comorbidity subgroups.

## 5. Conclusions

In summary, our results indicated that a limited set of routinely collected laboratory inflammatory parameters at admission could be useful for in risk stratification for prediction of disease severity and mortality in COVID-19 hospitalized patients. Biomarkers panels were superior to single biomarkers for predicting the severity and outcomes in patients with COVID-19. The main findings of our study strengthen the hypothesis that common indicators of dysregulated inflammation collected at admission can better predict mortality because the mixed pathophysiology nature of the COVID-19 patients caused by the coexistence of multiple comorbidities is slightly reflected at admission. Considering this significant heterogeneity, the identification of comorbid conditions with a common pathophysiology may result in improved prognosis for COVID-19 patients.

In conclusion, our results demonstrate that, in the future, diverse prediction models will play an essential role in the early risk stratification, monitoring, and tailoring of treatment of COVID-19 patients in order to improve outcomes.

## Figures and Tables

**Table 1:** Baseline demographic characteristics and clinical features on admission of the patients with COVID-19.

	Missing Data	All Patients (*n* = 254)
**Characteristics**		
**Ethnicity (*n*,%)**		
Romanian		254(100%)
**Sex (*n*, %)**		
Male		141 (55.5%)
Female		113 (44.5%)
**Age** (years, Mean ± S.D)		56.20 ± 17.13 56 (43.75–71)
**Smoking history**	1	
Yes		103 (40.6%)
No		150 (49.1%)
**Laboratory parameters**		
WBC (×10^9^/L)	1	8.71 ± 6.69 6.42 (4.6–10.36)
LYM (×10^9^/L)	0	1.68 ± 2.18 1.31 (0.92–1.89)
PLT (×10^9^/L)		247.17 ± 113.61 231 (168.75–300.25)
HGB (g/dL)		13.23 ± 3.77 13.3 (12.18–14.3)
NEU (×10^9^/L)		6.7 ± 7.38 4.16 (2.63–8.05)
NLR		6.88 ±9.63 3.24 (1.55–8.1)
PLR		209.81 ± 144.19 162.31 (118.06–272.74)
SII		1547.12 ± 1957.88 697.92 (330.81–2013.48)
ESR (mm/h)		49.53 ± 71.25 40 (15–72)
APTT (s)	2	31.73 ± 10.37 30.45 (28–32.8)
D-Dimer (ng/mL)		1284.1 ± 4761.84 245.5 (139–591.25)
CRP (mg/L)	1	52.89 ± 72.48 18.03 (3.52–84.67)
ALT (U/L)		44.46 ± 58.05 32 (19–53)
AST (U/L)		49.39 ± 109.17 32 (21–47.25)
Total bilirubin (mg/dL)	4	0.74 ± 2.14 0.53 (0.36–0.78)
LDH (U/L)		236.14 ± 205.73 199 (129.25–314.25)
CK (U/L)	42	177.28 ± 327.78 84 (56–141)
Serum creatinine (mg/dL)		1.35 ± 5.82 0.72 (0.57–0.98)
Blood urea nitrogen (mg/dL)		53.83 ± 55.46 36.5 (26–58)
Ferritin (ug/L)	28	496.08 ± 548.42 326.5 (142–632)
**Onset symptoms**		
Fever		151 (59.4%)
Cough		156 (61.4%)
Sputum production		46 (18.1%)
Fatigue		112 (44.1%)
Dizziness		65 (25.6%)
Diarrhea		25 (9.8%)
Headache		90 (35.4%)
Anorexia		28 (11%)
Dyspnea		131 (51.6%)
Nausea		10 (3.9%)
Shortness of Breath		99 (39%)
**Baseline Vital Signs**		
Temperature, °C	44	37.91 ± 0.92 38 (37.3–38.63)
BMP	1	86.01 ± 19.95 87 (78–97)
SAO_2_	1	91.6 ± 11.24 95 (89.5–98)
**Length of hospitalization (days)**	3	15.06 ± 7.22 14 (10–20)
**Comorbidities**		
Hypertension	1	140 (55.1%)
Diabetes	1	62 (24.4%)
COPD	1	61 (24%)
Cardiovascular diseases	1	71 (28%)
Liver disease	1	19 (7.5%)
Renal disease without hemodialysis	1	26 (10.2%)
Obesity	1	54 (21.3%)
Cancer	1	28 (11%)
Cerebrovascular disease	1	29 (11.4%)
**Complications**		
ARDS		41 (16.1%)
Liver dysfunction		20 (7.9%)
Acute kidney injury		14 (5.5%)
Pneumonia		151 (59.4%)
Septic shock	1	13 (5.1%)
**Oxygen therapy**		
No O_2_ requirement		136 (53.5%)
O_2_ by face mask or nasal prongs		62 (24.4%)
Noninvasive mechanical ventilation		13 (5.1%)
Invasive mechanical ventilation		39 (15.4%)
**Antiviral treatment**		
Yes		59 (23.2%)
No		195 (76.8%)
**Antibiotic Treatment**		
Yes		185 (72.8%)
No		69 (27.2%)
**Imaging findings**		
Ground glass opacities		117 (46.1%)
Consolidation		53 (20.9%)
Pleural effusion		5 (2.0%)
Bronchiectasis		10 (3.9%)
Emphysema		4 (1.6%)
No lesions		73 (28.7%)
**Hospitalization outcome**		
Survived		184 (72.4)
Death		70 (27.6)

**Table 2:** Differences between levels of laboratory variables at admission and clinical severity (Mild, Moderate, Severe, Critical).

Mean ± S.D					*p* Value				
Median (IQR)									
					Mann–Whitney U test				
Laboratory Test	**Mild**	**Moderate**	**Severe**	**Critical**	Mild vs. Severe	Moderate vs. Severe	Mild vs. Critical	Moderate vs. Critical	Critical vs. Severe
	**(*n* = 85)**	**(*n* = 98)**	**(*n* = 34)**	**(*n* = 37)**					
Age (years)	47.38 ± 17.03	55.1 ± 16.0	65.7 ± 15.3	67.59 ± 12.53	<0.0001	0.001	<0.0001	<0.0001	0.5702
	44.5 (34.8–55.8)	53 (44–68.3)	66.5 (56.8–77)	70 (58.5–78)					
Male	45 (52.9%)	52 (53.1%)	22 (64.7%)	22 (59.5%)	0.9999	0.3163	0.6781	0.5639	0.8071
Female	40 (47.1%)	46 (46.9%)	12 (35.3%)	15 (40.5%)					
**Laboratory parameters**									
WBC (×10^9^/L)	5.8 ± 2.9	7.9 ± 6.2	13.3 ± 9.5	12.45 ± 7.18	<0.0001	0.0007	<0.0001	<0.0001	0.8976
	5.2 (4.1–6.8)	6.1 (4.6–9.3)	10.8 (5.8–18.3)	10.7 (7.2–17.7)					
LYM (×10^9^/L)	1.8 ± 0.9	1.6 ± 1.1	1.4 ± 2.1	1.13 ± 0.65	<0.0001	<0.0001	<0.0001	0.0033	0.2227
	1.6 (1.2–2.3)	1.3 (0.9–1.9)	0.8 (0.6–1.1)	0.9 (0.7–1.3)					
PLT (×10^9^/L)	231.1 ± 89.2	266.5 ± 121.8	224.4 ± 114.5	234.08 ± 134	0.5863	0.0881	0.5805	0.0998	0.9932
	208 (169.3–275.8)	247 (186.8–323.3)	214.5 (136.3–274.8)	200 (131–330.5)					
HGB (g/dL)	14.2 ± 4.2	13.4 ± 4.3	12.6 ± 2.7	11.83 ± 2.37	0.0531	0.6985	<0.0001	0.0034	0.0631
	13.8 (12.9–14.9)	13.3 (12.3–14.3)	13.2 (11.7–14.2)	12.4 (10.3–13.4)					
NEU (×10^9^/L)	3.4 ± 2.7	6.3 ± 8.5	11.3 ± 8.7	10.63 ± 6.6	<0.0001	<0.0001	<0.0001	<0.0001	0.808
	2.87 (1.9–4.1)	3.9 (2.6–7.6)	8.7 (4.4–16.1)	9.2 (6–15.1)					
NLR	2.4 ± 2.2	5.3 ± 7.2	16.3 ± 15.5	12.65 ± 10.51	<0.0001	<0.0001	<0.0001	<0.0001	0.5049
	1.7 (1.03–2.9)	2.9 (1.6–6.8)	12.4 (5.2–23.3)	10 (5.8–16.7)					
PLR	147.7 ± 71.41	220.9 ± 158.5	273.1 ± 146.7	263.7 ± 175.9	<0.0001	0.0083	0.0006	0.1777	0.4761
	132.6(91.4–168.3)	166.6 (119.2–277.7)	249.8 (178.3–330.1)	224.7 (123.8–362.7)					
SII	574.3 ± 766.6	1440.4 ± 1788.9	2900.9 ± 2307.2	2745.7 ± 2576.5	<0.0001	<0.0001	<0.0001	<0.0001	0.7123
	382.4 (186.8–634.8)	688.8 (360–2016.6)	2238.2 (1099.5–4521.1)	1845.4 (971.4–3588.2)					
ESR (mm/h)	31.5 ± 30.4	57.1 ± 106.1	54.6 ± 30.6	62.7 ± 31.9	<0.0001	0.0576	<0.0001	0.001	0.1214
	17 (10–49.3)	39.5 (20–76.3)	54.5 (37.8–83.3)	70 (41.5–90)					
APTT (sec)	31.9 ± 5.7	29.8 ± 3.6	32.9 ± 11.9	35.9 ± 22.3	0.1315	0.9024	0.4056	0.1394	0.2828
	32 (29.3–33.2)	29.6 (27.3–32.1)	29.2 (25.9–35.2)	30 (28.1–33)					
D-Dimer (ng/mL)	302.04 ± 516.6	408.8 ± 537.5	2624.4 ± 7118.4	4634.2 ± 9695.0	<0.0001	0.0001	<0.0001	<0.0001	0.13
	145 (100.5–224.8)	241 (143.8–410.3)	528.5 (231.8–1441.5)	679 (368.5–3781)					
CRP (mg/L)	28.9 ± 64.1	47.1 ± 64.7	87.7 ± 77.6	96.9 ± 80.9	<0.0001	<0.0001	<0.0001	<0.0001	0.8661
	5.9 (2.03–25.8)	16.5 (3.8–79.4)	56.6 (27.6–147.9)	87.9 (32.4–129.5)					
ALT (U/L)	37.4 ± 29.6	54.5 ± 84.7	41.5 ± 38.4	38.9 ± 26.2	0.748	0.2576	0.5684	0.4068	0.8035
	28 (17.3–50)	36 (20.8–57)	31 (19.3–54.3)	31 (18.5–54)					
AST (U/L)	31.7 ± 18.8	51.6 ± 89.6	81.8 ± 250.6	55.3 ± 45.1	0.0572	0.9157	0.0167	0.4067	0.4653
	26 (21–37)	32.5 (22.8–51.8)	34.5 (21.8–58.3)	38 (22.5–88)					
Total Bilirubin (mg/dL)	0.6 ± 0.3	0.7 ± 1.1	0.6 ± 0.5	1.5 ± 5.3	0.1835	0.8555	0.7848	0.5515	0.5474
	0.5 (0.4–0.8)	0.6 (0.4–0.8)	0.6 (0.4–0.8)	0.6 (0.4–0.8)					
LDH (U/L)	164.3 ± 82.1	223.4 ± 150.2	381.2 ± 341.3	310.2 ± 260.6	<0.0001	<0.0001	<0.0001	<0.0001	0.874
	165 (122–207)	203 (143–276.5)	327 (219.8–504.8)	325 (0.1–501.8)					
CK (U/L)	124.8 ± 181.5	172.4 ± 311.2	208.3 ± 359.7	347.7 ± 606.4	0.3736	0.8622	0.2022	0.8712	0.7179
	72 (53.3–116.8)	100 (59.5–159.5)	87 (54.8–169.5)	88 (55–366)					
Serum Creatinine (mg/dL)	0.7 ± 0.2	0.9 ± 0.7	0.9 ± 0.7	4.3 ± 15.0	0.1625	0.68	<0.0001	0.001	0.0447
	0.7 (0.6–0.8)	0.7 (0.6–0.9)	0.7 (0.5–1.2)	0.9 (0.7–1.9)					
Blood Urea Nitrogen (mg/dL)	32.8 ± 12.3	45.9 ± 50.4	76.4 ± 54.3	101.3 ± 87.7	<0.0001	0.0001	<0.0001	<0.0001	0.3062
	30 (25–38.5)	34 (24–53.3)	58 (36.8–110.3)	68 (40–125.5)					
Ferritin (ug/L)	263.8 ± 311.7	474.8 ± 531.6	812.6 ± 646.4	735.5 ± 596.3	<0.0001	0.0009	<0.0001	0.0002	0.7671
	198 (56–368)	337 (142–599)	630.5 (328.5–1162.3)	589 (395–764)					

**Table 3:** Logistic regression analysis of the models of predictors for severity (models 1–3) and mortality (models 4–8).

Model	Predictors for Severity	*p*-Value from Hosmer–Lemeshow Test	Accuracy	AUC (95% CI)	*p*-Value from Predicted Probability
Model1	Age, D-Dimer, LDH	0.854	79%	0.84 (0.78–0.89)	<0.0001
Model2	D-Dimer, APTT, CK, Total bilirubin, Creatinine, BUN	0.048	83%	0.69 (0.58–0.79)	0.005
Model3	D-Dimer, Creatinine, BUN	0.134	81%	0.85 (0.79–0.9)	<0.0001
**Model**	**Predictors for mortality**	***p*-value from Hosmer–Lemeshow Test**	**Accuracy**	**AUC (95% CI)**	***p*-value from predicted probability**
Model4	Age, SII, APTT	0.278	77%	0.83 (0.77–0.88)	<0.0001
Model5	D-Dimer, APTT, CK, Total bilirubin, Creatinine, BUN	0.63	82%	0.81 (0.74–0.89)	<0.0001
Model6	D-Dimer, BUN	0.013	78%	0.82 (0.76–0.88)	<0.0001
Model7	CRP, Ferittin, NEU, NLR, SII, PLR	0.005	81%	0.86 (0.81–0.91)	<0.0001
Model8	NEU, NLR, SII, PLR	0.257	78%	0.85 (0.8–0.9)	<0.0001

## Data Availability

The datasets generated and analyzed during the study are available from the corresponding authors upon reasonable request.

## Supplementary Materials

The online version contains supplementary material that can be downloaded at: https://www.mdpi.com/article/10.3390/v14061201/s1. Table S1: The severity risk factors identified in this study using a logistic regression model included elderly age, higher D-Dimer and LDH levels, Table S2: Risk factors associated with death in COVID-19 patients, Table S3: Area Under the Curves for the severity of disease, Table S4: Area Under the Curves for mortality, Table S5: Differences between levels of laboratory variables at admission within the subgroups of comorbidities, survived vs. deceased COVID-19 patients.
