# Supplementary material for: Risk of Death in Comorbidity Subgroups of Hospitalized COVID-19 Patients Inferred by Routine Laboratory Markers of Systemic Inflammation on Admission: A Retrospective Study
Source: Viruses. 2022 May 31;14(6):1201. doi: 10.3390/v14061201 (PMC9228480; doi:10.3390/v14061201)
Supplement: Supplementary file 1 [file viruses-14-01201-s001.zip › viruses-1740724-supplementary.pdf]

Supplementary Material

Table S1 reports the severity risk factors identified in this study using a logistic regression model included elderly age, higher D-Dimer and LDH levels.

Table S2 reports the mortality risk factors identified in this study using a logistic regression model included elderly age, higher SII and APTT levels.

Table S3 reports the analysis of all AUCs for the single laboratory parameters and models predicting performance for disease severity.

Table S4 reports the analysis of all AUCs for the single laboratory parameters and models predicting performance for mortality.

Table S5 reports the differences of the routine laboratory parameters collected on admission among the subgroups of comorbidities, discharged and non-discharged COVID-19 patients.

Table S1. The severity risk factors identified in this study using a logistic regression model included elderly age, higher D-Dimer and LDH levels.

	Univariable OR (95% CI)	*p*-value	Multivariable OR (95% CI)	*p*-value
Age	1.04 (1.0-1.07)	0.05	1.05 (1.03-1.07)	**<0.0001**
WBC (x 10^9/L)	1.0 (0.99-1.00)	0.62	-	
LYM (x 10^9/L)	1.17 (0.78-1.74)	0.44	-	
PLT (x 10^9/L)	1.06 (0.77-1.48)	0.71	-	
HGB (g/dL)	0.99 (0.98-1.01)	0.39	-	
NEU (x 10^9/L)	0.98 (0.77-1.24)	0.86	-	
NLR	0.89 (0.57-1.42)	0.64	-	
PLR	1.14 (0.92-1.42)	0.23	-	
SII	1.0 (0.99-1.01)	0.58	-	
ESR (mm/h)	1.01 (0.98-1.03)	0.41	-	
APTT (sec)	1.03 (0.95-1.11)	0.46	-	
D-Dimer (ng/mL)	1.0 (1.0-1.001)	0.05	1.001 (1.0-1.001)	**<0.0001**
CRP (mg/L)	1.0 (0.99-1.01)	0.87	-	
ALT (U/L)	0.99 (0.97-1.02)	0.54	-	
AST (U/L)	0.99 (0.97-1.03)	0.97	-	
Total bilirubin (mg/dL)	1.07 (0.28-4.01)	0.93	-	
LDH (U/L)	1.0 (1.0-1.01)	0.028	1.003 (1.001-1.01)	**<0.0001**
CK (U/L)	1.0 (0.99-1.0)	0.75	-	
Serum Creatinine (mg/dL)	0.98 (0.65-1.5)	0.94	-	
Blood Urea Nitrogen (mg/dL)	0.99 (0.99-1.01)	0.68	-	
Ferritin (ug/L)	1.0 (0.99-1.01)	0.73	-	

P-value: derived from Mann-Whitney U-test; Significant differences are shown in bold ($p < 0.0001$).

Table S2. Risk factors associated with death in COVID-19 patients

	Univariable OR (95% CI)	*p*-value	Multivariable OR (95% CI)	*p*-value
Age	1.06 (1.02-1.10)	0.004	1.05 (1.03-1.07)	**<0.0001**
WBC (x 10^9/L)	1.43 (0.89-2.29)	0.131	-	
LYM (x 10^9/L)	1.0 (0.63-1.59)	0.988	-	
PLT (x 10^9/L)	0.99 (0.98-1.0)	0.212	-	
HGB (g/dL)	0.84 (0.67-1.07)	0.158	-	
NEU (x 10^9/L)	1.26 (0.68-2.33)	0.461	-	
NLR	1.11 (0.86-1.42)	0.434	-	
PLR	1.01 (1.0-1.02)	0.004	1.0 (0.99-1.0)	0.854
SII	0.99 (0.99-1.0)	0.008	1.0 (1.0-1.001)	**<0.0001**
ESR (mm/h)	0.99 (0.97-1.02)	0.542	-	
APTT (sec)	1.08 (0.99-1.16)	0.062	1.05 (1.01-1.09)	0.02
D-Dimer (ng/mL)	1.0 (1.0-1.0)	0.898	-	
CRP (mg/L)	1.0 (0.99-1.01)	0.350	-	
ALT (U/L)	0.98 (0.96-1.01)	0.117	-	
AST (U/L)	1.02 (0.98-1.05)	0.298	-	
Total bilirubin (mg/dL)	1.49 (0.39-5.77)	0.563	-	
LDH (U/L)	1.0 (0.98-1.0)	0.918	-	
CK (U/L)	1.0 (0.99-1.0)	0.882	-	
Serum Creatinine (mg/dL)	2.86 (0.52-15.79)	0.227	-	
Blood Urea Nitrogen (mg/dL)	0.98 (0.96-1.01)	0.163	-	
Ferritin (ug/L)	1.0 (0.99-1.0)	0.836	-	

P-value: derived from Mann-Whitney U-test; Significant differences are shown in bold ($p < 0.0001$).

Table S3. Area Under the Curves for the severity of disease.

Parameters	AUC (95% CI)	p-value
Age	0.77 (0.71-0.84)	**< 0.0001**
WBC	0.74 (0.66-0.83)	**< 0.0001**
NEU	0.80 (0.73-0.88)	**< 0.0001**
NLR	0.84 (0.77-0.9)	**< 0.0001**
PLR	0.68 (0.58-0.77)	**< 0.0001**
SII	0.78 (0.71-0.86)	**< 0.0001**
ESR	0.68 (0.59-0.76)	**< 0.0001**
DDimer	0.81 (0.74-0.88)	**< 0.0001**
CRP	0.75 (0.67-0.84)	**< 0.0001**
AST	0.57 (0.47-0.67)	0.14
TOTAL bilirubin	0.51 (0.41-0.61)	0.86
LDH	0.79 (0.71-0.87)	**< 0.0001**
CK	0.51 (0.41-0.61)	0.81
Creatinine	0.57 (0.46-0.67)	0.17
Urea	0.75 (0.67-0.83)	**< 0.0001**
Ferritin	0.78 (0.71-0.85)	**< 0.0001**
ALT	0.51 (0.43-0.59)	0.75
APTT	0.51 (0.42-0.59)	0.82
HGB	0.63 (0.55-0.71)	0.001
PLT	0.58 (0.49-0.66)	0.067
LYM	0.74 (0.67-0.82)	**< 0.0001**
Model1 (Age, D-Dimer, LDH)	0.84 (0.78-0.89)	**< 0.0001**
Model2 (D-Dimer, APTT, CK, Total bilirubin, Creatinine, BUN)	0.69 (0.58-0.79)	0.005
Model3 (D-Dimer, Creatinine, BUN)	0.85 (0.79-0.91)	**< 0.0001**

P-value: derived from Mann-Whitney U-test; Significant differences are shown in bold (p < 0.0001).

Table S4. Area Under the Curves for mortality

Parameters	AUC (95% CI)	p-value
Age	0.79 (0.74-0.85)	**< 0.0001**
WBC	0.74 (0.65-0.83)	**< 0.0001**
NEU	0.79 (0.73-0.87)	**< 0.0001**
NLR	0.85 (0.79-0.91)	**< 0.0001**
PLR	0.69 (0.61-0.78)	**< 0.0001**
SII	0.8 (0.74-0.87)	**< 0.0001**
ESR	0.63 (0.54-0.71)	0.007
DDimer	0.77 (0.69-0.85)	**< 0.0001**
CRP	0.76 (0.69-0.84)	**< 0.0001**
AST	0.56 (0.47-0.66)	0.175
TOTAL bilirubin	0.59 (0.5-0.68)	0.055
LDH	0.72 (0.63-0.81)	**< 0.0001**
CK	0.56 (0.47-0.65)	0.203
Creatinine	0.64 (0.54-0.73)	0.004
Urea	0.76 (0.68-0.85)	**< 0.0001**
Ferritin	0.73 (0.65-0.8)	**< 0.0001**
ALT	0.53 (0.45-0.61)	0.04
APTT	0.52 (0.43-0.6)	0.043
HGB	0.65 (0.57-0.73)	0.04
PLT	0.56 (0.47-0.65)	0.044
LYM	0.72 (0.65-0.79)	**< 0.0001**
Model4 (Age, SII, APTT)	0.83 (0.77-0.88)	**< 0.0001**
Model5 (D-Dimer, APTT, CK, Total bilirubin, Creatinine, BUN)	0.81 (0.74-0.89)	**< 0.0001**
Model6 (D-Dimer, BUN)	0.82 (0.76-0.88)	**< 0.0001**
Model7 (CRP, Ferittin, NEU, NLR, SII, PLR)	0.86 (0.81-0.91)	**< 0.0001**
Model8 (NEU, NLR, SII, PLR)	0.85 (0.8-0.9)	**< 0.0001**

P-value: derived from Mann-Whitney U-test; Significant differences are shown in bold ($p < 0.0001$).

Table S5. Differences between levels of laboratory variables at admission within the subgroups of comorbidities, survived vs. deceased COVID-19 patients.

	**Deceased (Yes, No)**																		**P value >>>>**								
	**Hypertension**		**Diabetes**		**COPD**		**Cardiovascular diseases**		**Liver diseases**		**Renal diseases**		**Obesity**		**Cancer**		**Cerebrovascular diseases**		Hypertension	Diabetes	COPD	Cardio	Liver	Renal	Obesity	Cancer	Cerebro vascular
	No (N=89)	Yes (n=51)	No (n=30)	Yes (n=32)	No (n=29)	Yes (n=32)	No (n=30)	Yes (n=41)	No (n=10)	Yes (n=9)	No (n=11)	Yes (n=15)	No (n=39)	Yes (n=15)	No (n=17)	Yes (n=11)	No (n=11)	Yes (n=18)	0.0003	**<0.0001**	0.0001	**<0.0001**	0.0582	0.0007	>0.9999	0.1751	**<0.0001**
Age (years, Mean ± S.D)	60.6±15.4	68.9±10.9	58.1±12.3	67.0±11.9	61.9±13.4	66.7±12.1	65.6±16.2	90.9±11.1	46.4±13.6	69±16.1	67.1±17.3	71.9±10.8	54±15.3	67.5±13.7	60.7±11.9	62.9±11.9	72.5±17.7	69.3±13.9	0.0011	0.0055	0.1632	0.2151	0.0141	0.6182	0.0038	0.6021	0.4180
Male	54 (60.7%)	30 (58.8%)	21 (70%)	16 (50%)	23 (71.9%)	18 (62.1%)	20 (66.7%)	24 (58.5%)	8 (80%)	6 (66.7%)	7 (63.6%)	7 (46.7%)	24 (61.5%)	5 (33.3%)	11 (64.7%)	8 (72.7%)	7 (63.6%)	11 (61.1%)	0.8591	0.1275	0.5856	0.6216	0.6285	0.4527	0.0759	>0.9999	>0.9999
Female	35 (39.3%)	21 (41.2%)	9 (30%)	16 (50%)	9 (28.1%)	11 (37.9%)	10 (33.3%)	17 (41.5%)	2 (2%)	3 (33.3%)	4 (36.4%)	8 (53.3%)	15 (38.5%)	10 (66.7%)	6 (35.3%)	3 (27.3%)	4 (36.4%)	7 (38.9%)									
**Laboratory parameters**																											
WBC (x 10^9/L)	7.3±4.2	13.3±9.8	8.3±5.2	14.3±9.9	8.5±5.7	14.4±8.9	8.8±4.9	14.2±10.0	8.3±6.8	16.6±15.2	8.6±7.7	16.2±12.6	7±4.1	13.8±6.6	10.5±6.8	11.9±4.6	6.7±4.4	14.8±12.3	**<0.0001**	0.0017	0.0080	0.02	0.2224	0.0237	**<0.0001**	0.2211	0.0214
LYM (x 10^9/L)	1.7±1.4	1.3±1.3	1.9±1.8	1.3±0.7	1.6±1.0	1.5±1.7	1.8±1.6	1.3±1.5	2.1±1.9	0.9±0.8	1.4±0.7	1.1±0.6	1.7±1.4	1±0.5	2.0±2.0	1.3±0.8	1.3±0.6	1.1±0.8	0.0003	0.0136	0.0242	0.0261	0.0789	0.0893	0.0039	0.2300	0.2247
PLT (x 10^9/L)	260.3±111.8	249.3±128.2	264.9±101.6	264.3±120.1	270.1±140.1	257.1±140.3	266.3±114.8	235.2±125.8	182.1±81.7	229.9±169.7	239.5±87.6	311.1±157.6	245.8±82.3	265.3±135.9	263.0±148.9	329.2±179.5	250.7±93.6	228.4±143.1	0.3210	0.6318	0.6182	0.2069	0.6038	0.1679	0.9886	0.4372	0.3687
HGB (g/dL)	13.1±1.9	12.2±2.6	13.3±2.3	12.3±2.3	13.1±2.5	11.9±2.6	12.7±2.4	12.1±2.4	12.9±1.6	10.3±3.6	12.6±2.6	11.1±3.0	13.9±2	11.6±2.7	12.1±2.7	12.1±3.8	12.6±0.9	12.4±2.6	0.0337	0.0336	0.0877	0.3645	0.0531	0.1935	0.0008	0.6024	0.6027
NEU (x 10^9/L)	5.1±3.8	11.1±8.9	5.9±4.8	12.0±9.0	8.1±12.0	10.2±8.1	6.5±4.7	11.9±9.1	11.9±20.3	14.8±13.8	6.3±6.9	13.8±11.3	4.9±3.9	11.6±5.4	7.7±6.3	9.5±3.4	4.7±4.6	12.5±11.3	**<0.0001**	**0.0001**	0.0058	0.0022	0.2775	0.0077	**<0.0001**	0.1755	0.0045
NLR	4.2±4.3	11.8±9.4	4.5±4.9	11.2±8.1	6.2±10.3	10.2±6.8	5.4±5.6	14.4±13.3	9.9±17.4	25.5±23.2	4.9±5.9	12.9±6.8	3.9±4.1	13.8±10.5	5.6±5.1	9.3±4.8	5±7.3	14.7±12.1	**<0.0001**	**<0.0001**	0.0007	**<0.0001**	0.0133	0.0005	**<0.0001**	0.0735	0.0125
PLR	199.3±127.9	276.9±171.9	186.5±121.4	258.6±148.6	189.4±100.1	276.7±195.1	195.2±98.0	265.1±154.1	128.3±80.8	359.3±231.3	191.8±97.0	322.1±188.2	181±91.4	289.3±172.2	167.9±91.5	316.3±218.8	220.1±115.1	264.1±175.8	0.0025	0.0246	0.0864	0.0328	0.0133	0.0473	0.0193	0.0326	0.5501
SII	1193.3±1621.9	2873.1±2435.4	1374.2±2039.3	2946.8±2360.9	1530.9±2086.5	2791.3±2460.7	1417.2±1647.9	3109.8±2611.4	1138±1803.5	3834.4±2607.5	1597.8±2798.6	3969.4±2956.8	1078.7±1644.7	3694.7±3045.9	1573.9±1841.7	3241.9±2895.9	1412.5±2446.1	3350.4±3003.9	**<0.0001**	**0.0001**	0.0104	0.0018	0.0172	0.0030	**<0.0001**	0.0417	0.0682
ESR (mm/h)	61.4±110.4	60.7±28.9	52.3±32.7	68.6±26.8	50.9±34.3	66.2±25.5	80.9±183.1	59.2±28.5	46.9±35.1	59.2±44	46.7±24.5	64±29.1	51.8±32.8	68.5±33.6	47.9±34.6	59.9±26.4	43.8±30.8	63.5±31.5	0.0319	0.0120	0.0796	0.1197	0.4839	0.1443	0.0883	0.2582	0.0893
APTT (sec)	30.2±4.9	30.2±7.8	29.9±6.3	28.8±6.3	31.2±4.1	30.1±9.7	29.6±6.0	30.8±9.7	31.7±3.0	45.1±31.0	29.1±4.7	29.1±4.2	30.9±5.9	29.9±5.5	31.4±6.0	26.9±9.6	31.1±4.3	29.4±3.5	0.8947	0.6978	0.0849	0.5771	0.2871	>0.9999	0.1962	0.2298	0.1696
D-Dimer (ng/mL)	754.1±2220.2	2480.9±7057.4	1370.0±3704.1	2018.2±5527.2	1132.3±3343.4	1775.1±5642.7	846.9±1685.9	3472.0±9083.5	779.4±1053.3	3244.6±5331.3	827.4±1069.3	808.5±1000.6	518.9±1381.8	1436.6±1777.3	1865.1±4542.9	1584.6±3980.9	722.7±820.4	3246.7±7228.7	0.0004	0.0051	0.0786	0.0578	0.2037	0.3914	**<0.0001**	0.7020	0.2758
CRP (mg/L)	40.2±53.9	99.2±75.9	39.6±54.6	108.8±87.9	45.8±86.2	103.8±82.7	57.0±73.6	87.5±69.9	59.7±88.2	96.4±79.1	23.8±26.5	101.5±92.5	51.4±58.7	116.1±91.1	53.6±108.5	84.3±75.1	70.3±79.3	80.6±64	**<0.0001**	0.0003	**<0.0001**	0.0132	0.0764	0.0016	0.0031	0.0427	0.3869
ALT (U/L)	44.3±39.9	35.9±26.2	38.1±21.8	34.5±24.4	43.2±57.1	40.0±32.6	32.4±20.3	35.1±24.8	114.1±226.2	43.7±58.5	29.5±21.7	29.9±15.9	43±25.9	32.1±13.9	47.5±56.5	21.9±16.6	34.7±24.5	41.3±33.2	0.1375	0.2723	0.5733	0.8373	0.2184	0.6368	0.2357	0.1568	0.6987
AST (U/L)	43.1±33.9	44.1±31.1	37.1±19.6	50.5±36.6	42.8±43.4	44.6±32.2	37.9±33.5	43.2±29.9	118.3±265.1	197.7±487	35.6±15.9	41.8±22.7	37.6±19.1	51.5±24.9	39.9±37.9	27.8±24.9	50.4±51.6	46.4±35.1	0.8199	0.2601	0.4750	0.50	0.9863	0.5826	0.0570	0.0793	0.8509
Total bilirubin (mg/dL)	0.6±0.4	1.3±4.5	0.6±0.5	1.7±5.6	0.6±0.4	1.8±5.8	0.6±0.3	0.7±0.5	1.4±3.5	4.5±11.1	0.5±0.2	2.9±8.4	0.6±0.4	2.7±8.1	0.6±0.5	3.7±9.4	0.5±0.3	2.4±7.4	0.0933	0.0837	0.2333	0.3581	0.2825	0.0771	0.2318	0.0540	0.0988
LDH (U/L)	229.1±148.9	314.9±214.9	235.2±148.7	278.9±218.6	217.5±144.3	298.6±228.8	257.3±160.1	324.2±207.8	150.4±135.5	415.8±549.2	213.3±178.4	320.4±240.9	212.9±126.4	390.4±277.5	224.9±145.5	190.3±150.4	234±186.9	222.8±149.9	0.0057	0.3482	0.1780	0.1441	0.1384	0.3227	0.0110	0.8804	0.7988
CK (U/L)	144.8±237.8	116.1±133.1	127.5±148.5	150.3±258.8	131.9±119.7	104.6±64.7	105.5±73.3	144.8±197.5	226.2±290.5	291±542.2	169.9±200.1	117.6±35.7	170.1±222.3	488.8±798.3	101.9±125.5	119.6±103.8	139.2±192.7	164±216	0.7650	0.5428	0.8108	0.6015	0.8071	0.7305	0.2295	0.3268	0.3154
Serum Creatinine (mg/dL)	0.9±0.5	3.4±12.8	0.9±0.7	4.5±16.2	0.9±0.7	4.5±16.7	0.9±0.5	1.9±3.2	0.7±0.2	11.8±29.7	1.1±1.1	7.9±23.0	0.8±0.6	7±23.2	0.7±0.3	9.6±27	0.9±0.7	7.3±21.4	0.0153	0.2206	0.1792	0.1015	0.0232	0.0666	0.0072	0.1432	0.1560
Blood Urea Nitrogen (mg/dL)	46.9±44.0	85.9±69.5	54.8±58.6	78.1±56.0	51.2±56.8	86.4±82.1	62.9±54.7	101.2±86.2	46.9±29.4	136.3±90.8	71.3±92.5	131.3±96.9	44.1±51.6	74.3±33.9	50.5±50.4	88±103.8	57.8±60.9	88.7±72.3	**<0.0001**	0.0026	0.0097	0.0128	0.0509	0.0052	**<0.0001**	0.3115	0.0814
Ferritin (ug/L)	385.7±270.0	727.1±584.1	499.7±447.3	849.1±676.9	394.3±372.4	661.4±554.9	379.4±246.6	704.0±569.5	735.4±1047.5	628±477.1	278.1±223.5	640.5±692.2	443.3±432.1	812.7±742.9	589.9±652.9	407.3±208.1	349.6±192.6	701.6±469.4	**<0.0001**	0.0259	0.0181	0.0028	0.2909	0.0552	0.0452	0.8291	0.0124

P-value: derived from Mann-Whitney U-test; Significant differences are shown in bold (p < 0.0001).
